# Clinical and molecular study of patients with thyroid dyshormogenesis and variants in the *thyroglobulin* gene

**DOI:** 10.3389/fendo.2024.1367808

**Published:** 2024-07-08

**Authors:** Mónica Fernández-Cancio, María Antolín, María Clemente, Ariadna Campos-Martorell, Eduard Mogas, Noelia Baz-Redón, Jordi Leno-Colorado, Gemma Comas-Armangué, Elena García-Arumí, Laura Soler-Colomer, Núria González-Llorens, Núria Camats-Tarruella, Diego Yeste

**Affiliations:** ^1^ Growth and Development group, Vall d’Hebron Research Institute (VHIR), Hospital Universitari Vall d’Hebron, Barcelona, Spain; ^2^ Centre for Biomedical Network Research on Rare Diseases (CIBERER), Instituto de Salud Carlos III (ISCIII), Madrid, Spain; ^3^ Department of Clinical and Molecular Genetics and Rare Disease, Hospital Universitari Vall d’Hebron, Barcelona, Spain; ^4^ Medicine Genetics Group, Vall d'Hebron Research Institute (VHIR), Hospital Universitari Vall d’Hebron, Barcelona, Spain; ^5^ Pediatric Endocrinology Section, Hospital Universitari Vall d’Hebron, Barcelona, Spain; ^6^ Pediatrics, Obstetrics and Gynecology and Preventive Medicine Department, Universitat Autònoma de Barcelona, Bellaterra, Spain; ^7^ Research Group on Neuromuscular and Mitochondrial Diseases, Vall d’Hebron Research Institute, Hospital Universitari Vall d’Hebron, Barcelona, Spain

**Keywords:** congenital hypothyroidism, thyroid dyshormonogenesis, thyroglobulin, *TG*, gene variant

## Abstract

**Introduction:**

Defects in any thyroid hormone synthesis steps cause thyroid dyshormonogenesis (THD). THD due to *thyroglobulin* (*TG*) gene variants is a cause of congenital hypothyroidism (CH) with a wide clinical spectrum, ranging from mild to severe permanent hypothyroidism. We present high-throughput sequencing results of patients with *TG* variants.

**Methods:**

A CH high-throughput sequencing-panel of the main genes involved in the regulation of thyroid hormonogenesis was performed to identify those *TG* variants that may be related to patient THD phenotype.

**Results:**

We identified 21 *TG* gene variants in 19 patients (11.8%) which could explain their phenotype. Ten of those (47.6%) were not previously described. CH was biochemically severe in these 19 patients. Eight of them were reevaluated after one month of discontinuing LT4 treatment and all had severe permanent hypothyroidism. We also identified another 16 patients who presented heterozygous *TG* variants, of whom, at reevaluation, five had mild permanent and only one had severe permanent hypothyroidisms.

**Discussions:**

In this study, 10 novel and 11 previously reported variants in the *TG* gene have been identified that could explain the phenotype of 19 patients from non-consanguineous families from a large THD cohort. Although not all these TG gene variants can explain all the patients’ THD phenotypes, some of them had severe or mild permanent hypothyroidism at reevaluation.

## Introduction

Primary congenital hypothyroidism (CH) is the most common endocrine disorder in neonates and infants with an incidence of one in 3,000 newborns (the female to male ratio is 2:1) (1–5) and is also one of the most common preventable causes of intellectual disability. If untreated, CH causes permanent intellectual disability and slow growth. Most CH patients can be detected by newborn screening programs during the first week after birth, when there may be no specific clinical symptoms or signs suggestive of CH.

Genetic classification divides CH into two main categories according to their causes: a) thyroid gland development disorders (the dysembryogenesis or thyroid dysgenesis group), which occurs in 60% of all CH patients or b) defects in any of the steps of thyroid hormone synthesis (the dyshormonogenesis group) in the remaining cases (6, 7). Thyroid dyshormonogenesis (THD) can be caused by different defects in genes involved in thyroid hormone biosynthesis. There are several well-known associated genes, including *DUOX1, DUOX2, DUOXA1, DUOXA2, SLC26A4, SLC26A7, SLC5A5, TPO, IYD and TG* (3, 6, 8–10).

Dyshormonogenesis due to thyroglobulin (*TG*) gene variants is a cause of CH with an estimated incidence of 1 in 67,000 to 1 in 100,000 newborns (11–15), although this incidence may be biased and is likely to change with the use of high-throughput sequencing. Their clinical spectrum ranges from euthyroid through mild or severe permanent hypothyroidism with high iodide uptake, negative perchlorate discharge test (PDT), low levels of serum TG, high levels of serum thyroid-stimulating hormone (TSH), and simultaneous low serum total tetraiodothyronine (thyroxine, T4) levels accompanied by low or normal serum triiodothyronine (T3) levels. In untreated patients, *TG* defects due to loss-of-function variants (frameshift, nonsense and splice site variants) or missense variants involving wild-type cysteine residues and the acetylcholinesterase-homology (ChEL) domain cause persistent thyroid hormone (TH) deficiency, characterized by a severe phenotype resulting in intellectual disability with a large goiter (10).

The human *TG* gene is a single copy gene of 268 Kb that maps on chromosome 8q24 and is divided into 48 exons, encoding a mRNA sequence of 8,455 nucleotides (nt) (of which 8,304 nt correspond to coding sequences, NCBI: NM_003235.5) (13, 15–19). The monomeric human TG preprotein has a leader peptide of 19 amino acids followed by a 2749-amino acid polypeptide (NP_003226.4) (18). To date, about 230 human *TG* gene variants associated with THD have been identified (10). Since THD is due to pathogenic variants in *TG* inherited in an autosomal recessive manner, the patients should be homozygous or compound heterozygous.

In this study, high-throughput sequencing of patients from a Spanish cohort with THD reveal the presence of 19 patients with *TG* variants. To the best of our knowledge, this cohort study is the largest in Spain with regard to the number of genes analyzed and patients with THD enrolled, about whom an extensive clinical description and a genotype-phenotype correlation have been made.

## Materials and methods

### Patients

Patients were diagnosed within the CH Neonatal Screening Program in Catalonia, based on TSH levels, and were treated at the Pediatric Endocrinology Unit of the Vall d’Hebron University Hospital (VUVH) between 2001 and 2022. This study is an ambispective, retrospective and longitudinal study.

All patients, or their responsible adults, provided informed consent for the use of their samples in research studies. Their confidentiality was maintained by assigning a sample code. Our project was approved by the Clinical Research Ethics Committee (CEIC) of the VHUH (PR (AMI) 390/2016).

The inclusion criteria were: serum TSH levels higher than 10 mIU/L (AutoDELFIA®), in a sample drawn at the time of diagnostic confirmation of CH, and with scintigraphic and/or ultrasound studies suggestive of THD. Patients with serum TSH levels below 10 mIU/L, or with thyroid agenesis or ectopia diagnosed by thyroid scintigraphy and/or ultrasound, or with recognized syndromes were excluded. Additional information on the possible existence of thyroid disease in members of the family or associated malformations/diseases was collected in all cases.

Serum TSH levels at confirmation were determined by electrochemiluminescence immunoassay (TSH3-Ultra, ADVIA Centaur®), free T4 (fT4) levels were determined by electrochemiluminescence immunoassay (FT4, ADVIA Centaur®), and TG levels were determined by electrochemiluminescence immunoassay (Elecsys® Tg II-Cobas®).

Permanent or transient CH was determined using results of thyroid function tests after temporary withdrawal of LT4 therapy at approximately 3 years of age. One month after discontinuing levothyroxine (LT4) treatment, TSH and fT4 levels were measured in venous blood sample. Individuals who showed continuous dependency on LT4 were diagnosed with permanent CH. After reevaluation, patients with TSH≥10 mIU/L and fT4<0.8 ng/dL were classified as severe permanent THD and patients with TSH≥10 mIU/L and fT4> 0.8ng/dL as mild permanent THD. Subsequently, these children were then repeatedly evaluated at regular intervals of six months to monitor thyroid function. Those who did not need continuous LT4 therapy were diagnosed with transient CH (TSH<5 mIU/L and fT4>0.8 ng/dL). Reevaluation was not carried out when LT4 requirements (>3.4 mcg/day) and genetic results were suggestive of TG deficiency.

The data describing clinical characteristics and anthropometric neonatal parameters are summarized in **Supplementary Table S1**.

### Genetic analysis

Peripheral blood DNA was extracted by automatic magnetic extraction (Chemagic™ 360, Perkin-Elmer, Waltham, MA, USA) and DNA concentration was measured by Qubit 2.0 fluorometer (Qubit dsDNA BR Assay; Thermo Fisher, Waltham, MA, USA).

To perform the genetic study, a CH high-throughput sequencing-panel was designed with the GeneRead DNAseq Custom Builder tool from Qiagen (https://www.qiagen.com/en/shop/genes-and-pathways/custom-products/custom-array-products/generead-designer/) which included the main genes involved in the regulation of thyroid hormonogenesis: *DUOX2, DUOXA2, IYD, PAX8, TG, TPO, TSHR, SLC26A4 and SLC5A5*. This CH panel consisted of 393 amplicons (with an average size of 194 base pairs, and coverage ±10 nt of the exon-intronic junction zone) and allows sequencing of exons and adjacent intronic gene regions of interest, with a total coverage of 96.4% of the target sequence (GeneRead, Qiagen, Hilden, Germany) (20). The GeneRead CH panel was performed on the DNA samples of the patients following the provider’s instructions. In summary, amplification by PCR, indexing and library preparation (NEBNext Ultra DNA library prep kit, New England Biolabs) of the DNA samples were carried out. In those cases of greater clinical complexity, samples were analysed with a customised targeted Seq-Cap EZ HyperCap (NimbleGen) designed to capture the following genes *ANO1, CDCA8, DUOX1, DUOX2, DUOXA2, FOXE1, GLIS3, GNAS, IYD, NKX2-1, NKX2-5, PAX8, SLC26A4, SLC5A5, TG, THRB, TPO* and *TSHR*.

Finally, library sequencing was performed in a MiSeq platform (Illumina, San Diego, CA, USA) and bioinformatic analysis of the obtained data was performed with MiSeq Control Software (MCS), MiSeq Reporter (Illumina Inc, San Diego CA, USA), GeneRead SeqVariant Analysis software (Qiagen) and ANNOVAR adapted to our own pipeline. Firstly, overrepresented sequences were trimming using Trimmomatic (21), removing the sequences corresponding to the cut adapters used. Afterwards, sequences were mapped against the GRCh37 reference genome, using BWA (22). Variant calling was performed with Genome Analysis Toolkit (GATK) (23), and the variant annotation was done using ANNOVAR (24). Sanger sequencing was performed to confirm detected candidate variants and to analyze target regions with low coverage (<20).

The classification of the variants was performed following the American College of Medical Genetics and Genomics (ACMG) criteria (https://www.acmg.net/) using Varsome (https://varsome.com/) (25). Prediction of the effect of variants identified in the splicing regions was evaluated by Alamut Visual 2.11 (https://www.interactive-biosoftware.com/es/alamut-visual/). Family co-segregation analysis was carried out, when possible. The nucleotide position in human *TG* mRNA was designated according to reference sequence (NM_003235.5). Variant nomenclature is according to the guidelines of the Human Genome Variation Society (HGVS) (https://www.hgvs.org/) (26).

The frequency and the functional annotation of the identified variants were checked in public databases (GnomAD, ExAC, 1000 Genome project). Genetic variants including frameshift, missense, nonsense, and splicing-site with a minor allele frequency (MAF) <0.01 identified in known dyshormonogenesis genes were considered for the analysis. If available, segregation analysis results were taken into consideration.

### *In silico* analysis

The sequence of TG (NP_003226.4) was aligned with other TG homolog peptide sequences found in the HomoloGene NCBI database (https://www.ncbi.nlm.nih.gov/homologene/).

### Statistical analysis

Results are expressed as mean ± SD for values that followed a normal distribution or median (range) for values that did not follow a normal distribution. Differences for the variables were calculated using non-parametric Kruskal-Wallis test. All analyses were carried out with the statistical program “R” [(R version 3.6.1; 2019-07-05), Copyright (C) 2015 The R Foundation for Statistical Computing], with the support of the Biostatistics Unit of the Vall d’Hebron Research Institute (VHIR).

## Results

In this study we patients with *TG* variants of a Spanish cohort with THD. We identified 21 *TG* gene variants in 19 patients, which could explain their phenotype (17 compound heterozygotes and 2 homozygotes). Clinical characteristics, hormonal results, scintigraphs, ultrasounds, and perchlorate discharge test (PDT) of patients with THD and *TG* variants are presented in **Table 1**. As it is shown, there are no patients in this group with a follow-up classification of transient hypothyroidism; all of them suffered from severe hypothyroidism.

**Table 1:** Clinical and genetic characteristics of patients with confirmed thyroid dyshormonogenesis diagnosis by variants in the *TG* gene.

Patient	Sex	Thyroid function at diagnosis			Scintigraphy	Reevaluation state	PDT	TG cDNA Change (NM_003235.5)	TG Amino acid change (NP_003226.4)	Pathogenicity (ACMG)	Reported (HGMD)	Zygosity	Cosegregation
		TSH^c^ mIU/L	fT4^c^ ng/dL	TG^c^ ng/mL									
P1	M	375	0.63	0.2	normal	SP	negative	c.886C>T/c.5386C>T	p.Arg296*/p.Gln1796*	P/LP	rep/rep	HetComp	Fa/Mo
P2	M	352.6	0.4	0.2	increase	NR	NA	c.886C>T/c.1143delC	p.Arg296*/p.Tyr382Thrfs*20	P/P	rep/rep	HetComp	Mo/Fa
P4	F	75	1	NA	normal	SP	negative	c.7851C>G/c.8144G>C	p.Tyr2617*/p.Cys2715Ser	LP/LP	new/new	HetComp	Mo/Fa
P6	F	375	1	NA	normal	SP	negative	c.886C>T/c.4588C>T	p.Arg296*/p.Arg1530*	P/P	rep/rep	HetComp	Mo/Fa
P7^a^	F	75	0.4	35.9	normal	SP	NA	c.1351C>T/ c.3842G>A	p.Arg451*/p.Cys1281Tyr	LP/LP	rep/rep	HetComp	Mo/Fa
P7^b^	M	608	0.3	NA	increase	SP	NA	c.1351C>T/c.3842G>A	p.Arg451*/p.Cys1281Tyr	LP/LP	rep/rep	HetComp	Mo/Fa
P8^a^	M	150	0.5	0.2	increase	NR	NA	c.886C>T/c.7240G>A	p.Arg296*/p.Gly2414Arg	P/LP	rep/new	HetComp	Mo/Fa
P8^b^	M	486	0.4	2.6	normal	NR	NA	c.886C>T/c.7240G>A	p.Arg296*/p.Gly2414Arg	P/LP	rep/new	HetComp	Mo/Fa
P10	M	22	1.6	22.6	normal	SP	NA	c.5184C>A/c.6263-9877A>G	p.Cys1728*/ p.^d^	P/VUS	rep/new	HetComp	Fa/Mo
P11	F	550	0.5	0.2	normal	NR	NA	c.5386C>T/c.5386C>T	p.Gln1796*/p.Gln1796*	LP	rep	Homo	Mo/-(Fa NA)
P12	F	518	0.2	NA	normal	SP	negative	c.886C>T/c.6262+1delG	p.Arg296*/p.^d^	P/LikleyP	rep/rep	HetComp	Mo/Fa
P13	M	91.3	0.7	13.6	increase	NA	NA	c.886C>T/c.5173delC	p.Arg296*/p.Leu1725Phefs*28	P/LP	rep/rep	HetComp	Fa/Mo
P17	M	309	0.3	73.5	increase	SP	negative	c.886C>T/c.3790T>G	p.Arg296*/p.Cys1264Gly	P/LP	rep/new	HetComp	Mo/Fa
P19	M	375	0.3	0.2	normal	NR	NA	c.886C>T/c.1333C>T	p.Arg296*/p.Arg445*	P/LP	rep/new	HetComp	Mo/Fa
P20	F	835	NA	0.2	NA (goi)	NR	NA	c.5976-1G>A/c.7228G>C	Intronic/p.Ala2410Pro	LP/VUS	new/ new	HetComp	NA
P21	M	406	0.7	0.1	increase	NR	NA	c.886C>T/c.2976C>G	p.Arg296*/p.Tyr992*	P/LP	rep/new	HetComp	Mo/Fa
P30	F	34.8	0.3	NA	normal	NA	NA	c.886C>T/c.5864-1G>A	p.Arg296*/ p.^d^	P/LP	rep/rep	HetComp	Mo/Fa
P31	F	386	0.49	0.1	increase	NA	NA	c.886C>T/ c.886C>T	p.Arg296*/ p.Arg296*	P/P	rep/rep	Homo	Mo/Fa
P33	F	604.6	0.38	0.2	increase	NA	NA	c.4986_4987del/c.5686+1G>T	p.Cys1662*/ p.^d^	LP/P	new/rep	HetComp	NA
Median (range)		375 (22-835)	0.49 (0.2-1.6)	0.2 (0.1-73.5)									

F, female; M, male; NA, not available; goi, goiter probably due to late diagnosis of THD; NR, not required; T, transient; SP, severe permanent; MP, mild permanent; PDT, perchlorate discarge test; fT4, free thyroxine; TG, thyroglobulin; TSH, thyrotropin; P, pathogenic; VUS, variant of uncertain significance; LP, likely pathogenic; rep, reported; Mo, mother; Fa, father; hetcomp, compound heterozygous; homo, homozygous; ^a,b^siblings. ^d^Change in the protein has not been proven.

^c^Reference values: TSH (0.9-10 mIU/L), fT4 (1.33-2.57 ng/dL), TG (3.5-77 ng/mL). * of "TG Amino acid change" column refers to Ter/stop but in HGVS * is accepted (see https://hgvs-nomenclature.org/stable/recommendations/protein/substitution/).

**Figure 1:**
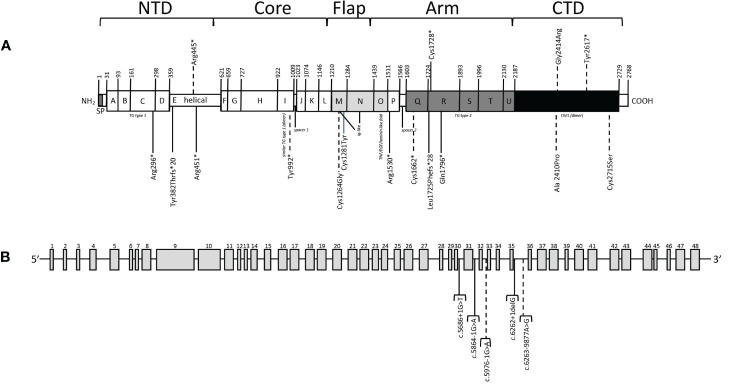
Variants detected in our patients. **(A)** Location of variants in primary TG structure. Thyroglobulin signal peptide (SP) and TG type 1 (the A, B, C, D, F, G, H, J, K, L and P domains), helical domain (E), TG type 3 (the Q, R, S, T and U domains), similar TG type 1 (dimer) (the I domain), Ig-like (the M and N domains), TNF/EGF/laminin-like fold (the O domain) and acetylcholinesterase-homology (ChEL (dimer), the V domain) domain types, are represented by boxes. The thyroglobulin monomer is organized in five structural regions: N-Terminal Domain (NTD), Core, Flap, Arm and C-Terminal Domain (CTD). The amino acid positions are numbered including the 19 amino acids of the signal peptide, following the NCBI: NP_003226.4 and Uniprot: P01266 numbering. **(B)** Location of intronic variants. Dashed lines indicate non reported variants. * refers to Ter/stop but in HGVS * is accepted (see https://hgvs-nomenclature.org/stable/recommendations/protein/substitution/).

A description of the 21 *TG* variants detected in our patients is included in **Table 2**. Four variants were located in the N-terminal domain (NTD), one in the core domain, two in the flap domain, ten in the arm domain and four in the carboxy-terminal domain (CTD). **Figure 1** shows the location of variants in the new primary TG structure described recently (10, 27, 28). Ten *TG* variants (47.6%) had not been previously described in the literature. Of these, six were classified as likely pathogenic and four as variants of uncertain significance (VUS) following ACMG guidelines.

**Table 2:** Thyroglobulin variants detected in patients with THD.

Intron/ Exon	cDNA Change NM_003235.5	Amino acid change NP_003226.4	dbSNP	Classical primary structure Domain/region^a^	New primary structure Domain type [domain]/region^b^	Mutation type	HGMD	Pathogenicity ACMG
Exon 7	c.886C>T	p.Arg296*	rs121912648	TG type 1-3/I	TG type 1 [C]/NTD	*nonsense*	Reported	P
Exon 9	c.1143delC	p.Tyr382Thrfs*20	rs778849740	Linker/I	Helical [E]/NTD	*frameshift*	Reported	P
	c.1333C>T	p.Arg445*	rs748309986	Linker/I	Helical [E]/NTD	*nonsense*	New	LP
	c.1351C>T	p.Arg451*	rs773142559	Linker/I	Helical [E]/NTD	*nonsense*	Reported	LP
Exon 11	c.2976C>G	p.Tyr992*	–	TG type 1-8/I	Similar TG type 1 dimer [I]/Core	*nonsense*	New	LP
Exon 17	c.3790T>G	p.Cys1264Gly	–	Hinge/I	Ig-like [M]/Flap	*missense*	New	LP
	c.3842G>A	p.Cys1281Tyr	–	Hinge/I	Ig-like [M]/Flap	*missense*	Reported	LP
Exon 22	c.4588C>T	p.Arg1530*	rs121912646	TG Type 1-11/II	TG type 1 [P]/Arm	*nonsense*	Reported	P
Exon 25	c.4986_4987del	p.Cys1662*	–	TG type 3a-1/III	TG type 1 [Q]/Arm	*frameshift*	New	LP
Exon 26	c.5173delC	p.Leu1725Phefs*28	rs1426751404	TG type 3b-1/III	TG type 3 [R]/Arm	*frameshift*	Reported	LP
	c.5184C>A	p.Cys1728*	rs199599591	TG type 3b-1/III	TG type 3 [R]/Arm	*nonsense*	Reported	P
Exon 27	c.5386C>T	p.Gln1796*	rs754658907	TG type 3b-1/III	TG type 3 [R]/Arm	*nonsense*	Reported	LP
Intron 30	c.5686+1G>T	–	rs374620255	TG type 3b-1/III	TG type 3 [R]/Arm	*splicing*	Reported	P
Intron 31	c.5864-1G>A	–	rs202044661	TG type 3a-2/III	TG type 3 [S]/Arm	*splicing*	Reported	LP
Intron 32	c.5976-1G>A	–	–	TG type 3a-2/III	TG type 3 [S]/Arm	*splicing*	New	LP
Intron 35	c.6262+1delG	skipping of exón 35	–	TG type 3b-2/III	Type 3 [T]/Arm	*splicing*	Reported	LP
	c.6263-9877A>G	–	rs17621034	TG type 3b-2/III	Type 3 [T]/Arm	*intronic*	New	VUS
Exon 41	c.7228G>C	p.Ala2410Pro	rs777769800	ChEL/IV	ChEL dimer [V]/CTD	*missense*	New	VUS
Exon 42	c.7240G>A	p.Gly2414Arg	–	ChEL/IV	ChEL dimer [V]/CTD	*missense*	New	LP
Exon 45	c.7851C>G	p.Tyr2617*	–	ChEL/IV	ChEL dimer [V]/CTD	*nonsense*	New	LP
Exon 47	c.8144G>C	p.Cys2715Ser	rs774585616	ChEL/IV	ChEL dimer [V]/CTD	*missense*	New	LP

The genomic position corresponds to the GRCh37 assembly. The nucleotide position is shown according to Homo sapiens thyroglobulin mRNA sequence in NCBI NM_003235.5. The amino acid positions are numbered including the 19 amino acids of the signal peptide, following the NCBI: NP_003226.4. HGMD, The Human Gene Mutation Database, https://www.hgmd.cf.ac.uk/ac/index.php; ACMG, American College of Medical Genetics and Genomics, https://www.acmg.net/; fs, frameshift; P, pathogenic; LP, likely pathogenic; VUS, variant of uncertain significance; ChEL, acetylcholinesterase-homology domain; NTD, N-terminal domain; CTD, C-terminal domain; ^a^Classical primary structure by Holzer et al. (15); ^b^New primary structure by Coscia et al. (16). * refers to Ter/stop but in HGVS * is accepted (see https://hgvs-nomenclature.org/stable/recommendations/protein/substitution/).

In this study, we also identified another 16 patients from the Spanish cohort who presented *TG* variants, which in themselves do not explain their THD classic phenotype. These cases were categorized into 3 groups: a) unconfirmed *TG* diagnosis: patients with heterozygous variants in *TG* gene (n=8); b) oligogenic group: patients with heterozygous variants in different related genes (n=5); and c) other genes: patients with monoallelic *TG* variant and homozygous or compound heterozygous variants in another gene that would explain their THD phenotype (n=3) (**Supplementary Table S2**).

### Clinical description

All patients were diagnosed with primary CH at birth by the Neonatal Screening Program in Catalonia followed by a venous-blood TSH assay for confirmation. Gestational age was 39.6 ± 2.3 weeks (mean ± SD), with a weight of 3299.9 ± 528.7 g and length of 49.5 ± 2.6 cm at birth. Only patient P20 was premature with a gestational age of 32 weeks, 1680 g and 41 cm at birth (**Supplementary Table S1**).

The median age at THD diagnosis was 14 (range 7-42) days after birth, with median and range TSH of 375 (22–835) mIU/L, fT4 of 0.49 (0.2-1.6) ng/dL and TG of 0.2 (0.1-73.5) ng/mL (**Table 1**).

Ten patients had normal thyroid uptake at THD diagnosis, eight had high iodide uptake, and goiter was evident at diagnosis in P20, who developed goiter probably because the diagnosis of THD was made late (42 days after birth) due to her prematurity and her late TSH peak. Of the 17 index cases, four had a family history of CH and two had an affected sibling (P7 and P8). A PDT was performed in five patients, all of whom had a negative result.

Eight out of 19 patients (42.1%) were reevaluated at approximately 3 years of age after one month of discontinuation of LT4 treatment. In addition, seven patients were not reevaluated because their LT4 requirements and genetic results were suggestive of permanent hypothyroidism, and in the remaining four data from reevaluation had not been obtained because they were less than 3 years old. All reevaluated patients had severe permanent hypothyroidism.

CH was more severe biochemically (p<0.05) in patients with confirmed molecular diagnosis (homozygous or compound heterozygous pathogenic variants in the TG gene), comparing TSH, fT4 and TG levels at diagnosis between patient groups (confirmed *TG* diagnosis group vs unconfirmed *TG* diagnosis and oligogenic groups) (**Table 1**). All patients with unconfirmed TG diagnosis were reevaluated, except P32; one had severe permanent hypothyroidism, three had mild permanent hypothyroidism and three had transient hypothyroidism. All patients of the oligogenic group, except P9, were reevaluated: two had mild permanent hypothyroidism and two transient hypothyroidism (**Supplementary Table S2**). Most of the patients in these groups had a high serum TG protein level and, at follow up, presented with a transient or mild phenotype, which is consistent with no *TG* pathogenic variant being clearly involved (only one affected allele or/and a VUS variant).

### Genetic analysis

High-throughput sequencing analysis identified 21 *TG* gene variants in 19 patients (17 index cases) with MAF <0.01 compared to public databases, and *in silico* prediction as pathogenic, likely pathogenic or VUS (**Table 2**).

Variants in homozygous state were identified in 2/19 patients (10.5%) and compound heterozygous variants in 17/19 (89.5%). These 19 patients showed a genotype-phenotype correlation and were considered diagnosed. These patients presented pathogenic [p.(Arg296*), p.(Tyr382Thrfs*20), p.(Arg1530*), p.(Cys1728*), c.5686 + 1G>T]; likely pathogenic [p.(Arg445*), p.(Arg451*), p.(Tyr992*), p.(Cys1662*), p.(Leu1725Phefs*28), p.(Gln1796*), c.5864-1G>A, c.5976-1G>A, c.6262 + 1delG, p.(Gly2414Arg), p.)Tyr2617*)]; or initially VUS [p.(Cys1264Gly), p.(Cys1281Tyr), p.(Cys2715Ser), c.6263-9877A>G, p.(Ala2410Pro)] *TG* variants. The presence of these *TG* variants could plausibly have contributed to the THD phenotype and cosegregation studies allowed the reclassification of p.(Cys1264Gly), p.(Cys1281Tyr) and p.(Cys2715Ser) missense variants as likely pathogenic. Family cosegregation studies have been carried out for 17 of the patients and, in all cases, the parents were heterozygous carriers of the detected variants (**Table 1**).

Variants detected in *TG* gene included missense variants (n=5), nonsense variants (n=8), frameshift deletions (n=3) and intronic variants (n=5), 4 of them in splicing nucleotides. Among the missense variants, four were initially VUS but only one remains classified as VUS, as segregation has been not possible. Furthermore, none of the four were previously described. Three of eight nonsense variants were not previously described and all of them were classified as pathogenic (n=3) or likely pathogenic (n=5) (**Table 2**).

Two of the frameshift variants, p.(Tyr382Thrfs*20) and p.(Leu1725Phefs*28), were previously described (29, 30), yet frameshift c.4986_4987del;p.(Cys1662*) variant was new (**Table 2**).

Three of the intronic variants affecting the canonical intronic splice site region, c.5686 + 1G>T, c.5864-1G>A and c.6262 + 1delG, were previously described (30, 31) and classified as pathogenic or likely pathogenic. The other intronic variant affecting the canonical intronic splice site, c.5976-1G>A, was novel and classified as likely pathogenic. In the other groups of patients, three of the four intronic variants observed were not previously described and classified as VUS (**Supplementary Table S3**), whereas the fourth is pathogenic and affects to the canonical site c.5975 + 5G>C.

The most frequent variant in the *TG* gene in our THD patient cohort was the pathogenic nonsense variant c.886C>T;p.(Arg296*) and is present in 12 of the 19 patients (63.2%).

Twenty-seven relatives (14 mothers, 13 fathers) of 17 different families were analyzed. *De novo* variants have not been detected in our cohort. Patient P20 is a result of an *in vitro* fecundation from sperm and oocyte donors, and the cosegregation study of P33 was not performed because the patient moved to another city. In both cases, we consider that *TG* variants are responsible for their THD phenotype, assuming that the two alleles are affected, since their clinical characteristics are consistent with pathogenic TG.

Eight patients had a non-confirmed diagnosis of THD due to TG defects (unconfirmed TG diagnosis group, **Supplementary Table S2**). Two of them (P5, P14) were compound heterozygous for *TG* variants, but with a maternal allele with a likely benign *TG* variant, following ACMG criteria. Moreover, according to the clinical and biochemical characteristics of these two patients, *TG* variants would not explain the phenotype. The other six patients of these group (P15, P16, P18, P22, P27, P32) were heterozygous for a VUS, likely pathogenic or pathogenic *TG* variant.

Three patients (P24, P28, P29; other gene group, **Supplementary Table S2**) were solved by compound heterozygous pathogenic *DUOX2* variants or the homozygous pathogenic variant in the *TPO* gene. Their phenotype and clinical characteristics correlated with variants in *DUOX2* and TPO, respectively.

### *In silico* analysis

Among the previously undescribed missense variants detected in our patients, a multiple sequence alignment of the human *TG* with sequences of other species revealed that wild-type cysteine residues at positions 1264 and 2715, wild-type alanine residue at position 2410, and wild-type glycine residue at position 2414 are strictly conserved in all *TG* species analyzed (**Supplementary Figure S1**), except residue 2410 that in *G. gallus* is a methionine. These four variants were detected in patients P17, P4, P20 and P8 siblings, respectively, in combination with pathogenic or likely pathogenic variants in the other allele.

## Discussion

In this study, we used a high-throughput sequencing panel with CH-related genes to perform a comprehensive genetic screening of patients with THD. All patients were diagnosed with primary CH at birth (serum TSH≥10 mUI/L) by the Neonatal Screening Program in Catalonia.

Dyshormonogenesis due to *TG* gene variants is a cause of CH and is inherited as an autosomal recessive trait. Only homozygous or compound heterozygous pathogenic variants in the *TG* gene were thought to be the cause of THD in our patients (“confirmed *TG* diagnosis” group). TSH, fT4 and TG values of this group at diagnosis are consistent with the molecular analysis. This finding confirms that very low TG levels are a good indication for *TG* gene variant screening [0.2 ng/mL (0.1-73.5) vs 219 ng/mL (10-1230) or 341 ng/mL (41.1-2031) in “unconfirmed TG diagnosis” or “oligogenic” groups, respectively]. The results of our study are in line with Targovnik et al. (32). In addition, genetically solved cases had a more severe phenotype than ambiguous cases at the reevaluation stage. However, the patients unsolved by pathogenic *TG* variants (unconfirmed TG diagnosis and oligogenic groups) included one case of severe permanent CH (P27) and five of mild permanent CH (P3, P14, P15, P16, P26) (**Supplementary Table S2**). In these cases, we assumed that these variants could coexist with an additional undetected copy number variation (CNV), intronic, or regulatory mutation in the *TG* gene or in another related gene that could explain the THD phenotype.

Our high-throughput sequencing analysis identified 21 different *TG* gene variants, ten (47.6%) of them not previously described. Of these, eight were classified as likely pathogenic and two as VUS. The pathogenicity of each variant was carefully evaluated based on clinical data including correlations with clinical phenotypes, hormonal results, previously published information, *in silico* prediction tool results, familiar cosegregation and location of variants in regions of interest of the TG protein.

The most frequent *TG* variant in our THD patient cohort (63.2%) was the pathogenic variant c.886C>T;p.(Arg296*). This variant was reported as the most frequent variant detected in Caucasian populations [originally published as p.(Arg277*)], taking homozygous, compound heterozygous or monoallelic variants (8, 10, 33–37). The functional consequence of p.(Arg296*) truncated protein is the complete loss of the central and carboxy-terminal hormonogenic domains, and consequently, limited ability to generate TH. However, p.(Arg296*) TG peptide retains its ability to synthesize T4 because it still harbors both the acceptor tyrosine24 and the donor tyrosine149. Affected cases usually have a CH phenotype (10, 38).

The presence of *TG* variants (pathogenic, likely pathogenic or VUS) in patients could plausibly be contributing to their THD (**Table 2**). Homozygous variants were identified in patients P11 and P31 [p.(Gln1796*) and p.(Arg296*), respectively], while compound heterozygous variants were identified in the rest of the patients. Twenty-one different *TG* variants were identified in this group of patients, ten of which were previously undescribed. Of these, likely pathogenic variant c.5976-1G>A affected the canonical intronic splice site, probably leading to aberrant splicing. Exon skipping in the *TG* gene can be caused by nucleotide substitutions in acceptor or donor splice sites involving -1 intronic position. Therefore, mRNA studies of patient P20 may help to clarify this. In addition, four undescribed variants were nonsense: p.(Arg445*), p.(Tyr992*), p.(Cys1662*) and p.(Tyr2617*). The deleterious effect of these nonsense variants is assumed, following the ACMG criteria (25), as variant loss of function (LOF) is a known mechanism of disease in the *TG* gene (pLoF 0.89) and they are not reported in population database (gnomAD v4.0.0). Moreover, they are predicted to undergo non-sense-mediated decay (NMD) because they are not located in the last exon or the last 50bp of the preliminary exon. Truncated proteins as a consequence of nonsense variants could represent adequate targets for the nonsense mediated mRNA decay (NMD) pathway, a known RNA surveillance mechanism that detects and then selectively and rapidly degrades mRNAs that contain premature terminated codons (39).

Three novel missense variants detected in these patients [p.(Cys1264Gly), p.(Cys2715Ser), p.(Ala2410Pro)] were initially classified as VUS, but the two first were reclassified as likely pathogenic after segregation. However, c.7240G>A p.(Gly2414Arg) is a variant which also affects the first nucleotide of the 42 exon and predicts a loosing of the acceptor site that may result in the skipping of the whole exon, which was why it was classified as likely pathogenic following ACMG guidelines. Multiple sequence alignment of human TG with sequences from other homologous species revealed that cysteine at positions 1264 and 2715, alanine at position 2410 and glycine residue at position 2414 are highly conserved (**Supplementary Figure S1**). Although familial segregation studies have helped in the interpretation of these missense novel genetic variants, functional *in vitro* studies or *in vivo* evidence of impaired TSH-stimulated mutant TG production will help to definitively confirm their pathogenicity (13). Such analyses have not been undertaken, but the novel identified variants were rare, conserved between species and segregated with phenotype and hormonal results, suggesting that in combination with another pathogenic *TG* allele it resulted in the THD phenotype.

Sequencing analysis of the *TG* gene revealed missense mutations, which were described involving cysteine residues, indicating that the loss of cysteine residues may eliminate disulfide bonds and alter the normal conformational structure of the TG (10, 32). The novel p.(Cys1264Gly) in patient P17 and p.(Cys2715Ser) in patient P4 could have remove the disulfide bond, altering the normal structure of TG, and in combination with nonsense variants p.(Arg296*) and p.(Tyr2617*), respectively, in the other alleles this may have led to CH.

Several missense mutations associated with CH were reported in the ChEL domain (32, 40), which is essential for the intracellular transport of TG to the site of its hormonogenesis via the secretory pathway (41–43). This domain is also required for protein dimerization and consequently plays a critical structural and functional role in the TG protein. It is well documented that pathogenic missense mutations in the ChEL domain can result in the intracellular retention of TG in the endoplasmic reticulum (ER) and premature degradation. This could be the explanation for the low TG levels in P8 siblings and P20, who were carriers of p.(Gly2414Arg) and p.(Ala2410Pro), respectively, in combination with a nonsense p.(Arg296*) and a splicing c.5976-1G>A variants in the other allele.

The previously described c.6262 + 1delG causes an inframe loss of exon 35 in the *TG* gene, generating a polypeptide that had lost 21 amino acids compared with the wild-type TG (31). The resulting protein is synthesized but cannot be released into the follicle lumen. Instead, this protein is retained and processed intracellularly. This variant was also present in a reported Galician family with several members who have congenital goitrous hypothyroidism caused by the same two variants detected in patient P12.

Three patients (P2, P13 and P33) carried frameshift variants, which were previously described (29, 30). Both P3 and P13 had the nonsense variant p.(Arg296*), therefore in these patients neither of the two alleles can synthesize a complete TG protein. The variant in patient P33 [p.(Cys662*)] was not previously described. Specifically, this variant changed two nucleotides of the codon for Cys, at the position of the deletion, for a stop codon.

Eight patients had a non-confirmed diagnosis of THD due to TG defects (unconfirmed TG diagnosis group, **Supplementary Table S2**). Two of them (P5, P14) were compound heterozygous for *TG* variants, but with a maternal allele with a likely benign *TG* variant, following ACMG criteria. Moreover, according to the clinical and biochemical characteristics of these two patients, *TG* variants would not explain the phenotype. The other six patients of these group (P15, P16, P18, P22, P27, P32) were heterozygous for a VUS, likely pathogenic or pathogenic *TG* variant. Previous reports of CH due to *TG* pathogenic variants most commonly involved biallelic mutations (4). Therefore, it is unclear whether the mild hypothyroidism of these patients after reevaluation was attributable to the monoallelic variants or whether they harbored another non-detected variant. With our panel gene we only detected exonic and flanking intronic regions, but not deep-intronic or regulatory variants. In addition, four of the variants presented in these unsolved patients [p.(Arg296*), p.(Arg1250Leu), p.(Gln1796*), p.(Arg2585Trp)] have been previously described as associated with THD but in homozygosis or compound heterozygosis (4). Indeed, the TSH, fT4 and TG values of patients of these unsolved groups at diagnosis were not consistent with the presence of a mutated TG protein. We assumed that these variants could coexist with an additional undetected CNV, intronic, or regulatory mutation in the *TG* gene or in another related gene that could explain this THD mild phenotype.

Interestingly, three patients (P24, P28, P29; other gene group, **Supplementary Table S2**) were solved by compound heterozygous pathogenic *DUOX2* variants or the homozygous pathogenic variant in the *TPO* gene. Their phenotype and clinical characteristics correlated with variants in *DUOX2* and TPO, respectively. In these 3 patients, the VUS *TG* variants may partially modify or not influence the phenotype of the patients.

Most cases with CH due to *TG* gene mutations show decreased serum TG levels before TH replacement and this is a key factor for the diagnosis of *TG* defects (34, 44). Genetic variants in the *TG* gene may result in structurally defective proteins severely impairing the functional ability of TG to serve as a matrix for T3 and T4 generation. Misfolded TGs may cause TG retention in the ER and premature degradation by the ER-associated protein degradation pathways, causing ER distention, and ER storage disease, although, it is likely that hydrolysis of limited amounts of mutated TG molecules may escape from the ER and migrate to the colloid, allowing synthesis of small amounts of the thyroid hormone (44). This is a possible mechanism providing a minimum of TH to the body, allowing detection of circulating levels of TG in patients P7a (35.6 ng/mL), P10 (22.6 ng/mL), P13 (13.6 ng/mL) and P17 (73.5 ng/mL). Other cases of *TG* mutations manifesting high TG serum levels have been reported (40, 45–47). These four patients were compound heterozygous carriers of a nonsense pathogenic variant in one allele, whereas in the other allele they harbored p.(Cys1281Tyr), c.6263-9877A>G, p.(Leu1725Phefs*28) and p.(Cys1264Gly) variants, respectively. We hypothesize that the mutated TG molecules produced by these variants could escape from ER, reach the follicular lumen and produce circulating TG levels in these patients.

With the widespread use of newborn screening programs and the application of improved molecular techniques, some THD cases have been genetically diagnosed (8, 9, 48–50), although the genetic heterogeneity of CH makes its diagnosis complex. To date, all reported genes with digenic variants are involved in the same metabolic pathway: thyroid hormone biosynthesis, and therefore oligogenicity, has often been proposed to underlie the intrafamilial variability seen in known genetic causes of CH, especially in association with *DUOX2* mutations (51). In our study, all oligogenic cases (n=5) (**Supplementary Table S2**) carried heterozygous *TG* variants in combination with heterozygous variants in three different genes: *IYD* (n=1), *DUOX2* (n=3) and *TPO* (n=1). This may indicate that iodide organification defects may be more common in CH patients, even though the interpretation of the dominant effect of some variants is controversial. For example, there have been monoallelic variants reported in *DUOX2* (52), and later this turned out to be associated with transient hypothyroidism (53, 54). In our six patients with oligogenicity, two presented mild permanent hypothyroidism, two presented a transient THD and two are awaiting reevaluation.

High-throughput sequencing has improved the detection of variants in candidate genes, therefore, their application after newborn screening and CH confirmation may be a valid strategy for rapidly obtaining an accurate diagnosis of CH due to THD. However, certain limitations were observed in our study. Firstly, 2/21 (9.5%) of *TG* variants detected in our patients were VUS, thus, *in vitro* functional studies would be necessary to confirm their pathogenicity. Secondly, our genetic analysis approach only detects single nucleotide substitution and short deletion/insertion of the coding region and 10-bp exon-intron boundaries of the nine CH causal genes analyzed. Therefore, we were unable to detect large CNVs, variants in other candidate genes, regulatory or deep intronic variants. To detect such variants, multiplex ligation-dependent probe amplification (MLPA) analysis, whole exome and/or whole genome sequencing should be performed, with the additional goal of identifying new candidate genes. Accordingly, we cannot discard the possibility that some of our monogenic “solved” cases carried additional variants in genes which were not screened in our study but could contribute to their CH phenotype.

In conclusion, we have identified 10 novel and 11 previously reported variants in the *TG* gene, in 19 Spanish patients from non-consanguineous families with CH due to THD. Although not all the variants found can explain the THD phenotype of patients, it is important to monitor them since some had mild to severe permanent hypothyroidism at reevaluation. This study contributes to the understanding of the molecular mechanisms involved in the development of dyshormonogenesis, and expands the clinical knowledge and evolution of patients with defective *TG*.

## Data availability statement

The datasets for this article are not publicly available due to concerns regarding participant/patient anonymity. Requests to access the datasets should be directed to the corresponding author.

## Ethics statement

The studies involving humans were approved by Clinical Research Ethics Committee of the VHUH [PR (AMI) 390/2016). The studies were conducted in accordance with the local legislation and institutional requirements. Written informed consent for participation in this study was provided by the participants’ legal guardians/next of kin. Written informed consent was obtained from the individual(s), and minor(s)’ legal guardian/next of kin, for the publication of any potentially identifiable images or data included in this article.

## Author contributions

MF-C: Writing – original draft, Writing – review & editing. MA: Writing – review & editing. MC: Writing – review & editing. AC-M: Writing – review & editing. EM: Writing – review & editing. NB-R: Writing – review & editing. JL-C: Writing – review & editing. GC-A: Writing – review & editing. EG-A: Writing – review & editing. LS-C: Writing – review & editing. NG-L: Writing – review & editing. NC-T: Writing – review & editing. DY: Writing – review & editing.

## Supplementary material

Supplementary Figure 1 Homology of TG from different animal species using NCBI database. Partial protein alignment are shown with new amino acid changes detected in our patients. The amino acids are indicated by the single-letter code. The amino acid sequences are based on GenBank protein database (*Homo sapiens* TG, NP_003226.4).

Supplementary Table 1 Clinical characteristics and anthropometric neonatal parameters of patients with confirmed thyroid dyshormonogenesis diagnosis due to variants in the *TG* gene.

Supplementary Table 2 Clinical and genetic characteristics of “unconfirmed *TG* diagnosis”, “oligogenic” and “other gene” groups of patients with *TG* variants.

Supplementary Table 3 Thyroglobulin variants of “unconfirmed TG diagnosis”, “oligogenic” and “other gene” groups of patients.
