# Supplementary material for: Clinical and molecular study of patients with thyroid dyshormogenesis and variants in the thyroglobulin gene
Source: Front Endocrinol (Lausanne). 2024 Jul 8;15:1367808. doi: 10.3389/fendo.2024.1367808 (PMC11260715; doi:10.3389/fendo.2024.1367808)
Supplement: Supplementary Figure 1 — Homology of TG from different animal species using NCBI database. Partial protein alignment are shown with new amino acid changes detected in our patients. The amino acids are indicated by the single-letter code. The amino acid sequences are based on GenBank protein database (Homo sapiens TG, NP_003226.4). [file Presentation_1.pptx]

## Slide 1
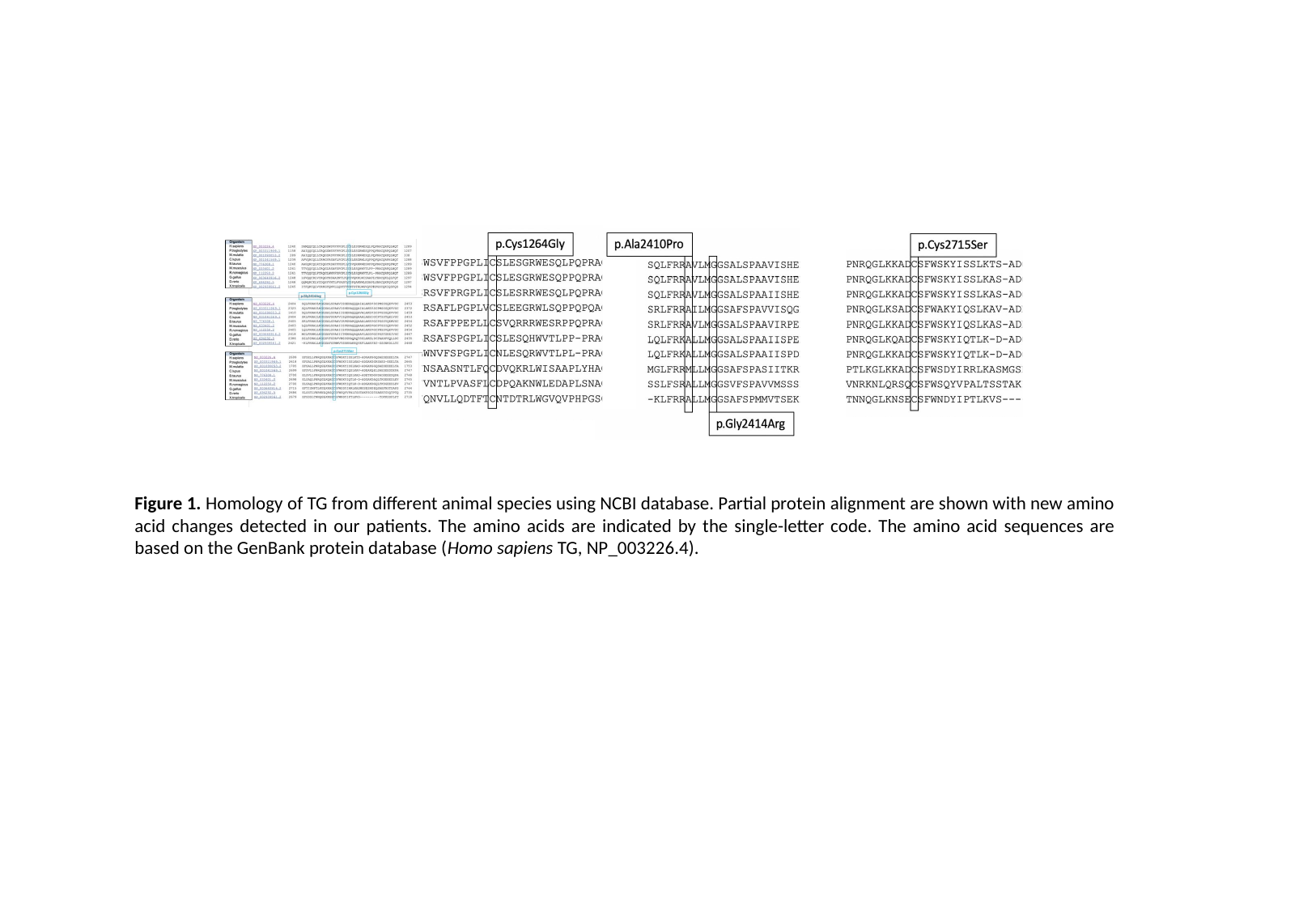

Figure 1. Homology of TG from different animal species using NCBI database. Partial protein alignment are shown with new amino acid changes detected in our patients. The amino acids are indicated by the single-letter code. The amino acid sequences are based on the GenBank protein database (Homo sapiens TG, NP_003226.4).
