# Supplementary material for: Clinical and molecular study of patients with thyroid dyshormogenesis and variants in the thyroglobulin gene
Source: Front Endocrinol (Lausanne). 2024 Jul 8;15:1367808. doi: 10.3389/fendo.2024.1367808 (PMC11260715; doi:10.3389/fendo.2024.1367808)
Supplement: Supplementary Table 1 — Clinical characteristics and anthropometric neonatal parameters of patients with confirmed thyroid dyshormonogenesis diagnosis due to variants in the TG gene. [file Table_1.docx]

**Supplemental Table 1**. Clinical characteristics and anthropometric neonatal parameters of patients with confirmed thyroid dyshormonogenesis diagnosis due to variants in the *TG* gene.

| **Patient** | **Sex** | **GA** | **Birth**  **Weight (g)** | **Birth**  **length**  **(cm)** | **Birth**  **length**  **(SD)** | **Family**  **History of CH** | **Age**  **diagnosis**  **(days)** |
| --- | --- | --- | --- | --- | --- | --- | --- |
|  |  |  |  |  |  |  |  |
| P1 | M | 41 | 3280 | 49.4 | -0.8 | NO | 14 |
| P2 | M | 41 | 3940 | 54 | 1.8 | NO | 14 |
| P4 | F | 42 | 3150 | 49.5 | -0.7 | NO | 16 |
| P6 | F | 40 | 3850 | 52 | 1.3 | NO | 17 |
| P7^a^ | F | 40 | 3490 | 49 | -0.5 | YES | 12 |
| P7^b^ | M | 40 | 3710 | 50 | -0.2 | YES | 14 |
| P8^a^ | M | 41 | 2790 | 50 | -0.4 | YES | 13 |
| P8^b^ | M | 42 | 3750 | 51 | -0.2 | YES | 12 |
| P10 | M | 40 | 3040 | 48 | -1.4 | NO | 11 |
| P11 | F | 39 | 3200 | 51 | 0.9 | NO | 14 |
| P12 | F | 38 | 3125 | 50 | 0.8 | NO | 13 |
| P13 | M | 40 | 3555 | 50 | -0.2 | YES | 13 |
| P17 | M | 41 | 4080 | 52 | 0.7 | NO | 27 |
| P19 | M | 37 | 3100 | 47 | -0.8 | YES | 15 |
| P20 | F | 32 | 1680 | 41 | -0.5 | YES | 42 |
| P21 | M | 39 | 2912 | 48 | -1.2 | NO | 7 |
| P30 | F | 38 | 3270 | 50 | 0.8 | NO | 18 |
| P31 | F | 40 | 3480 | 50 | 0.1 | YES | 11 |
| P33 | F | 41 | 3296 | 48 | -1.3 | NO | 9 |
| Descriptive statistics^c^ |  | 39.6±2.3 | 3299.9±528.7 | 49.5±2.6 | -0.2 (-1.4-1.8) |  | 14 (7-42) |

Abbreviations: F, female; M, male; GA, gestational age; ^a,b^siblings; ^c^median±SD for values that followed a normal distribution or median (range) for values that did not follow a normal distribution.

**Supplemental Table 2**. Clinical and genetic characteristics of “unconfirmed *TG* diagnosis”, “oligogenic” and “other gene” groups of patients with *TG* variants

| **Patient** | **Sex** | **Family**  **history** | **Thyroid function** | | | **Scintigraphy** | **Reevaluation state** | **PDT** | **LT4 treatment** | **TG cDNA Change**  **(NM_003235.5)** | **TG Amino acid change**  **(NP_003226.4)** | **Pathogenicity**  **(ACMG)** | **Reported (HGMD)** | **Zygosity** | **Cosegregation** |
| --- | --- | --- | --- | --- | --- | --- | --- | --- | --- | --- | --- | --- | --- | --- | --- |
|  |  |  | **TSH**  **mIU/L** | **fT4**  **ng/dL** | **TG**  **ng/mL** |  |  |  |  |  |  |  |  |  |  |
| **Unconfirmed *TG* diagnosis (*TG* non pathogenic variants or only heterozygous *TG* variants)** | | | | | | | | | | | | | | | |
| P5 | F | NO | 10 | 1.6 | 455 | normal | T | NA | NO | c.886C>T/c.6181G>A/  c.7753C>T | p.Arg296*/p.Gly2061Arg/ p.Arg2585Trp | P/LB/VUS | Rep/new/rep | HetComp | Fa/Mo/Fa |
| P14 | F | NO | 540 | 0.5 | 1230 | normal | MP | negative | YES | c.7813C>T/c.3804C>T | p.Arg2605*/p.Ser1268= | LikleyP/LikelyB | new/new | HetComp | Fa/Mo |
| P15 | M | YES | 46.7 | 1.1 | 159 | normal | MP | negative | YES | c.5767C>T | p.Pro1923Ser | VUS | new | Het | Fa |
| P16 | F | YES | 15 | 1.2 | 219 | decrease | MP | NA | YES | c.5401+49_5401+50delinsCG | p.? | VUS | new | Het | Mo |
| P18 | M | YES | 13.5 | 1.5 | 65.8 | decrease | T | NA | NO | c.3749G>T | p.Arg1250Leu | VUS | rep | Het | Mo |
| P22 | M | NO | 15.2 | 1.7 | 253.1 | decrease | T | NA | NO | c.886C>T | p.Arg296* | P | rep | Het | NA |
| P27 | M | YES | 178.3 | 0.9 | 523 | decrease | **SP** | NA | YES | c.5041+46C>G | p.? | VUS | new | Het | Mo |
| P32 | F | YES | 10,2 | 1.69 | 10 | normal | NA | NA | NO | c.5386C>T | p.Gln1796* | LP | rep | Het | NA |
| **Oligogenic group (heterozygous *TG* variants + heterozygous variants in another gene)** | | | | | | | | | | | | | | | |
| P3 | M | NO | 18 | 1.2 | 159 | decrease | MP | NA | YES | c.493G>T/c.5041+46C>T | p.Glu165*/p.? | LP/VUS | new/new | Het | Mo/Mo |
| P9 | M | NO | 83.1 | 0.9 | 1073 | increase | NA | NA | YES | c.416G>A | p.Trp139* | P | new | Het | NA |
| P23 | M | NO | 15 | 1.2 | 101 | decrease | T | negative | NO | c.886C>T | p.Arg296* | P | rep | Het | NA |
| P25 | F | NO | 90.2 | 1.9 | 41.1 | decrease | T | NA | NO | c.1267C>T | p.Arg423Cys | VUS | new | Het | NA |
| P26 | F | YES | 231 | 0.55 | 2031 | normal | MP | NA | YES | c.5975+5G>C | p.? | P | new | Het | NA |
| **Other gene (compound heterozygous/homozygous in another gene + heterozygous *TG* variants)** | | | | | | | | | | | | | | | |
| P24 | F | YES | 23.7 | 1.2 | 550 | increase | NA | NA | YES | c.4270T>G | p.Phe1424VaL | VUS | new | Het | Mo |
| P28 | F | NO | 348.6 | 0.1 | 1234 | NA (goiter) | NA | NA | YES | c.3795C>A/c.3847C>T/  c.6130C>T | p.Ser1265Arg/p.Arg1283Trp/  p.Arg2044Cys | VUS/VUS/VUS | new/new/rep | Het | NA |
| P29 | M | NO | 26.8 | 0.3 | NA | decrease | NA | NA | YES | c.3749G>T | p.Arg1250Leu | VUS | Rep | Het | NA |

Abbreviations: F, female; M, male; NA, not available; NR, not required; T, transient; SP, severe permanent; MP, mild permanent; PDT, perchlorate discarge test; fT4, free thyroxine; TG, thyroglobulin; TSH, thyrotropin. P, pathogenic; VUS, variant of uncertain significance; LP, likely pathogenic; rep, reported; Mo, mother; Fa, father; hetcomp, compound heterozygous; homo, homozygous.

**Supplemental Table 3.** Thyroglobulin variants of “unconfirmed TG diagnosis”, “oligogenic” and “other gene” groups of patients.

| **Intron/**  **Exon** | | **cDNA Change**  **NM_003235.5** | **Amino acid change**  **NP_003226.4** | **dbSNP** | **Classical primary structure Domain/region^a^** | **New primary structure**  **Domain type [domain]/region^b^** | **Mutation**  **type** | **Reported (HGMD)** | **Pathogenicity ACMG** |
| --- | --- | --- | --- | --- | --- | --- | --- | --- | --- |
|  | Exon 4 | c.416G>A | p.Trp139* | - | TG type 1-2/I | TG type 1 [B]/NTD | *nonsense* | New | P |
|  | Exon 5 | c.493G>T | p.Glu165* | rs1278276582 | TG type 1-3/I | TG type 1 [C]/NTD | *nonsense* | New | LP |
|  | Exon 9 | c.1267C>T | p.Arg423Cys | rs201039438 | Linker/I | Helical [E]/NTD | *missense* | New | VUS |
|  | Exon 17 | c.3749G>T | p.Arg1250Leu | rs114944116 | Hinge/I | Ig-like [M]/Flap | *missense* | Reported | VUS |
|  |  | c.3795C>A | p.Ser1265Arg | rs375533716 | Hinge/I | Ig-like [M]/Flap | *missense* | New | VUS |
|  |  | c.3804C>T | p.Ser1268= | rs61747461 | Hinge/I | Ig-like [M]/Flap | *synonymous* | New | LikelyB |
|  |  | c.3847C>T | p.Arg1283Trp | rs138431235 | Hinge/I | Ig-like [M]/Flap | *missense* | New | VUS |
|  | Exon 20 | c.4270T>G | p.Phe1424Val | rs776688425 | Hinge/I | Ig-like [M]/Flap | *missense* | New | VUS |
|  | Intron 25 | c.5041+46C>T | - | rs372960747 | TG type 3a-1/III | TG type 1 [Q]/Arm | *intronic* | New | VUS |
|  |  | c.5041+46C>G | - | rs372960747 | TG type 3a-1/III | TG type 1 [Q]/Arm | *intronic* | New | VUS |
|  | Intron 27 | c.5401+49_5401+50delGAinsCG | - | - | TG type 3b-1/III | TG type 3 [R]/Arm | *intronic* | New | VUS |
|  | Exon 31 | c.5767C>T | p.Pro1923Ser | rs577872355 | TG type 3a-2/III | TG type 3 [S]/Arm | *missense* | New | VUS |
|  | Intron 32 | c.5975+5G>C | - | - | TG type 3a-2/III | TG type 3 [S]/Arm | *intronic* | New | P |
|  | Exon 34 | c.6130C>T | p.Arg2044Cys | rs751735110 | TG type 3b-2/III | Type 3 [T]/Arm | *missense* | Reported | VUS |
|  |  | c.6181G>A | p.Gly2061Arg | rs115436575 | TG type 3b-2/III | Type 3 [T]/Arm | *missense* | New | LikelyB |
|  | Exon 44 | c.7753C>T | p.Arg2585Trp | rs114211101 | ChEL/IV | ChEL dimer [V]/CTD | *missense* | Reported | VUS |
|  | Exon 45 | c.7813C>T | p.Arg2605* | rs759171140 | ChEL/IV | ChEL dimer [V]/CTD | *nonsense* | New | LP |

The genomic position corresponds to the GRCh37 assembly. The nucleotide position is shown according to *Homo sapiens* thyroglobulin mRNA sequence in NCBI NM_003235.5The amino acid positions are numbered including the 19 amino acids of the signal peptide, following the NCBI: NP_003226.4. HGMD, The Human Gene Mutation Database, https://www.hgmd.cf.ac.uk/ac/index.php; ACMG, American College of Medical Genetics and Genomics, https://www.acmg.net/; fs, frameshift; P, pathogenic; LP, likely pathogenic; VUS, variant of uncertain significance; ChEL, acetylcholinesterase-homology domain; NTD, N-terminal domain; CTD, C-terminal domain; ^a^Classical primary structure by Holzer *et al.* (12); ^b^New primary structure by Coscia *et al.* (13).
